# Structure of the RNA-dependent RNA polymerase P2 from the cystovirus φ8

**DOI:** 10.1038/s41598-024-75213-7

**Published:** 2024-10-09

**Authors:** Merlyn Latimer-Smith, Paula S. Salgado, Ismay Forsyth, Eugene Makeyev, Minna M. Poranen, Dave I. Stuart, Jonathan M. Grimes, Kamel El Omari

**Affiliations:** 1 Diamond Light Source, Harwell Science and Innovation Campus, Didcot, OX110DE UK; 2 Division of Structural Biology, Wellcome Centre for Human Genetics, University of Oxford, Oxford, OX3 7BN UK; 3 Molecular and Integrative Biosciences Research Program, Faculty of Biological and Environmental Sciences, University of Helsinki, Helsinki, Finland; 4 Rutherford Appleton Laboratory, Research Complex at Harwell, Didcot, OX11 0FA UK; 5 Present Address: Biosciences Institute, Faculty of Medical Sciences, Newcastle University, Newcastle upon Tyne, UK; 6 Present Address: Centre for Developmental Neurobiology, Kings College London, London, SE1 1UL UK

**Keywords:** Cystovirus, RNA-dependent RNA polymerases, Crystallography, X-ray crystallography, Enzyme mechanisms, Viral proteins

## Abstract

The replication of RNA viruses relies on the activity of RNA-dependent RNA polymerases (RdRps). Despite large variations in their genomic sequences, viral RdRps share a common architecture generally known as a closed right hand. The P2 polymerase of cystovirus φ6 is currently among the best characterized viral RdRps. This polymerase is responsible for carrying out both replication and transcription of the viral double-stranded RNA genome using *de novo* initiation. Despite the extensive biochemical and structural studies conducted on φ6 P2, further structural information on other cystoviral RdRps is crucial to elucidate the structural and functional diversity of viral RdRps. Here, we have determined the atomic X-ray structure of the RdRp P2 from the φ6-related cystovirus φ8 at 3Å resolution. This structure completes the existing set of structural information on the φ8 polymerase complex and sheds light on the difference and similarities with related cystoviral RdRps.

## Introduction

Double-stranded RNA (dsRNA) viruses infect a variety of hosts, both eukaryotic and prokaryotic, and as such are highly diverse. dsRNA viruses tend to have segmented dsRNA genomes, which are transcribed and replicated by the viral RNA-dependent RNA polymerases (RdRps) typically confined in an icosahedral capsid shell. This multi-subunit assembly is often referred to as the polymerase complex^1^.

In most dsRNA viruses, RdRps perform both genome replication and transcription without the need for primers; during replication RdRps use positive-sense single-stranded RNA - (+)ssRNA - transcripts to produced dsRNA, whereas during transcription the produced negative-sense RNA - (-)ssRNA - serves as a template to produce (+)ssRNA copies from the dsRNA template. Studying this dual function of RdRps can be challenging and well-characterised dsRNA viruses, including those representing the *Cystoviridae* family, are used to elucidate key mechanistic features, as well as enzyme specificity, through biochemical and structural studies.

Cystoviruses are characterised by a genome consisting of three segments of linear dsRNA enclosed in an icosahedral polymerase complex that forms the virion core, further covered by an additional protein shell, and an envelope. The polymerase complex is made up of four proteins: P1, the major capsid protein; P2, the RdRp; P4, a packaging NTPase; and P7, an accessory protein^2^. Together these proteins are responsible for ensuring that the three dsRNA genomic segments are packaged into each virion. In the empty polymerase complexes (procapsids) of cystovirus φ6, the P2 RdRp is localized underneath the icosahedral three-fold symmetry axes, where it makes specific contacts with the P1 subunits at the neighbouring five-fold axes^3,4^and contributes to the conformational stability of the empty capsid^5^. P2 of φ6 and φ8 contribute to the procapsid self-assembly process likely via stabilization of interactions between P1 pentamers near the three-fold axis^5,6^.

The structure of P2 was initially determined from φ6 bacteriophage^7^, the archetypal cystovirus, and its template, substrate and divalent metal ion specificity have been comprehensively investigated by structural methods since then^8–11^.

The size varies significantly among RNA-dependent polymerases, and larger RdRps often contain additional functional domains^12^. φ6 P2 contains 665 amino acids, including a minimal RdRps domain consisting of approximately 400 amino acids, which makes φ6 P2 one of the smaller-sized RdRps. φ6 P2 has the canonical hand-like features of nucleic acid polymerases, with the main subdomains assigned to the fingers, thumb and palm as established by the crystal structure of the Klenow fragment of DNA polymerase^13,14^. φ6 P2 also has features unique to RdRps, such as the N-terminus of the fingers subdomain, the fingertips, that interacts with the thumb resulting in an encircled active site and an overall structure called the closed hand^15^. A second feature specific to RdRps is motif F, a nucleotide binding site^16^ and the conserved motif S/GDD involved in metal ion binding^17^. The φ6 P2 has two tunnels that allow the ssRNA template and nucleotide triphosphate substrate to reach the catalytic site^7–9^.

P2 from another cystovirus, φ12, has also been characterised using X-ray crystallography, which revealed an overall fold very similar to φ6 P2, despite a sequence identity of only ~ 21%^18^. Contrary to φ6 P2, the structure was reported in an inactive conformation that prevents formation of a stable initiation complex, with a flexible loop hindering the template access to the catalytic site.

Although cystoviral P2 RdRps catalyse the same reactions, they have distinct template specificities. For example, P2 of cystovirus φ8 prefers ssRNA templates with 3’-ends similar to those found in the φ8-specific minus strands^19^. These data suggest that P2 specificity may have coevolved with the (-)ssRNA 3’-proximal sequence to guarantee efficient transcription initiation.

To illuminate the differences between P2 from φ8 and its related cystoviruses, we have determined the structure of φ8 P2 by X-ray crystallography using an AlphaFold2 (AF2)-generated model for molecular replacement with PHASER, to 3Å resolution. The structure of φ8 P2, in addition to the previously determined structures of φ8 P1 and φ8 P4 (4BX4 and 4BWY, respectively), provide a more detailed view of the polymerase complex of φ8.

## Materials and methods

### Protein production and crystallisation

Expression of φ8 P2 was previously described^19^. Recombinant full-length P2 protein from φ8 was expressed from the plasmid pHY1 in *Escherichia coli* BL21 at 37 °C in LB medium containing 150 mg mL^−1^ of ampicillin until OD_540nm_ reached 0.6. Cultures were then diluted 50-fold, and incubation was maintained until the OD_540_ reached 1.0. Expression was induced with 1 mM isopropyl-β-d-thiogalactopyranoside (IPTG) followed by incubation overnight at 23 °C. Cells were harvested by centrifugation and lysed with a French pressure cell. φ8 P2 proteins were purified using Reactive Brown 10 agarose (Merck, Darmstadt, Germany) and a Superdex 75 gel filtration column (GE Healthcare, Little Chalfont, UK). φ8 P2 was concentrated to 10 mg mL^−1^ by ultracentrifugation for crystallisation studies.

Initial crystallization trials were performed by the sitting-drop vapor-diffusion method at 292 K using several Hampton and Emerald kits with a Cartesian technologies pipetting robot setting up 100 nL protein : 200 nL reservoir solution drops in Greiner 96-well plates^20^. Crystals of ɸ8 P2 appeared in 140 mM CaCl_2_, 14% PEG 3350, 100 mM Tris pH 8.

## Data collection, structure solution and refinement

Crystals of φ8 P2 were flash-cooled in liquid nitrogen using 20% glycerol as cryoprotectant. A 360° dataset was collected in August 2003 at a wavelength of 0.9393Å, from a single crystal at 100 K, on a CCD detector at the beamline ID14 at ESRF (Grenoble, France) with an oscillation range of 1° and exposure time of 1.5 s. Diffraction data to 3Å resolution was indexed in the *P*6_1_22 space group, integrated, scaled, and reduced with XIA2-DIALS^21,22^.

The crystal structure was solved by molecular replacement using PHASER^23^ with a search model predicted by AF2^24^. The PHASER solution, with one molecule in the asymmetric unit, was then subjected to rounds of manual rebuilding with COOT^25 ^and refinement with PHENIX.REFINE^26^ to a final R_work_/R_free_of 25.4/28.1%. The final model was validated with MOLPROBITY^27^. Data collection and refinement statistics can be found in Table 1. The structure factors and coordinates have been deposited in the PDB under the accession code 9FEJ.

**Table 1:** Data collection and refinement statistics. Values for the outer shell are given in parentheses.

	φ8 P2
Wavelength (Å)	0.9393
Resolution range (Å)	79.90–3.00 (3.10–3.00)
Space group	*P* 6_1_ 2 2
Unit cell (Å,°)	96.7 96.7 267.6 90 90 120
Total number of reflections	316,796 (23843)
Number of unique reflections	15,585 (1484)
Multiplicity	20.3 (16.1)
Completeness (%)	95.36 (57.77)
Mean I/sigma(I)	4.71 (0.23)
Wilson B-factor (Å^2^)	81.28
R-merge	0.2212 (1.891)
R-meas	0.2269 (1.953)
R-pim	0.0498 (0.474)
CC1/2	0.996 (0.690)
CC*	0.999 (0.904)
Reflections used in refinement	14,896 (877)
Reflections used for R-free	800 (43)
R-work	0.2544 (0.3924)
R-free	0.2807 (0.3971)
CC(work)	0.929 (0.540)
CC(free)	0.926 (0.736)
R.m.s.d. Bonds (Å) / Angles (°)	0.002 / 0.44
Ramachandran: Favoured/Allowed/Outliers (%)	96.67 / 3.33 / 0
Rotamer outliers (%)	1.87
Clashscore	3.9
Average B-factor (Å^2^)	79.5

## f’’ refinement

Refined f’’ values for the potential anomalous scatterers (Ca^2+^, Mg^2+^ and Mn^2+^) were obtained using PHENIX.REFINE^26^. Positional and B-factor refinement (xyz reciprocal and individual B-factors) were run first for five cycles, assuming Friedel’s law to be true. The B-factors of the metal ions were checked against the neighbouring atoms, and since they were of similar values, the output was used for the sole f’’ refinement, now assuming Friedel’s law to be false with occupancies fixed at one. f’ was set at the theoretical value for the ion and f’’ refined with a starting value of 0. Results can be found in Table 2.

**Table 2:** Refined, theoretical f’’ as well as B-factors values are reported for three independent refinements where the ion in the non-catalytic site was modelled as either calcium, magnesium and manganese ions.

	Ca^2+^	Mg^2+^	Mn^2+^
Refined f’’	0.54	0.70	0.59
Theoretical f’’	0.53	0.07	1.21
B-Factor (Mean B-Factor)	96.7 (79.5)	74.9 (70.0)	129.6 (78.8)

## Results and discussion

### Structure of φ8 P2

**Fig. 1:**
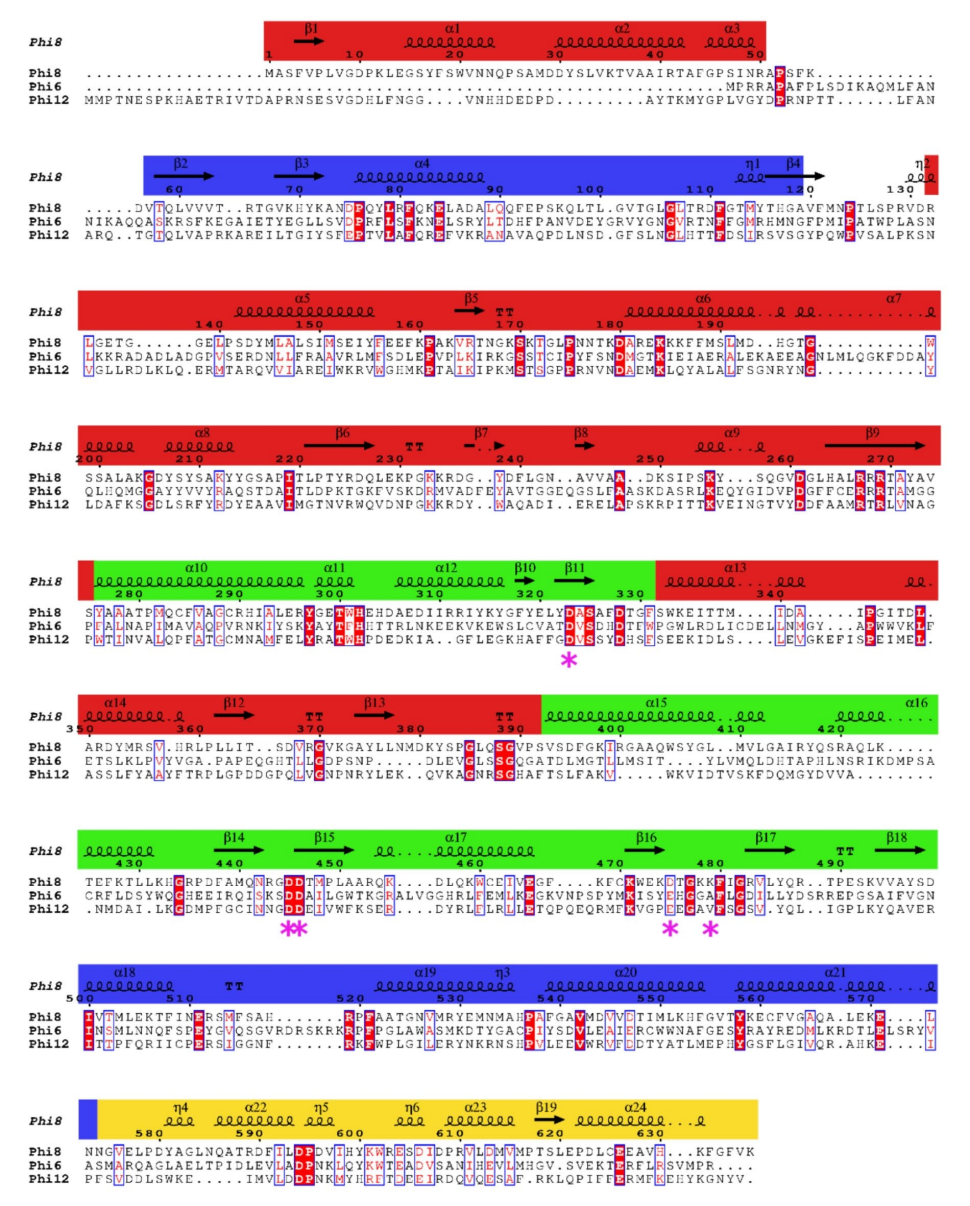
Sequence alignment of P2 RdRps from bacteriophages φ8, φ6 and φ12.The alignment was generated using Clustal Omega^46^. Sequence annotation was generated using the ESPript web service^47^. Conserved residues are highlighted in red while similar residues have a red-type colour and are surrounded by a blue outline. The pink asterisks indicate the residues interacting with metal ions. The secondary structure assignment for φ8 P2 is shown on top of the sequence and the fingers, palm, thumb and C-terminal subdomains coloured in red, green, blue and yellow respectively.

The full-length φ8 P2 structure (residues 2-636) was determined at 3Å resolution by molecular replacement using an AF2 generated search model. The structure of φ6 P2 (PDB 1HI8) and φ12 P2 (PDB 4IEG) are available in the PDB. These share ~ 18% and 23% sequence identity with φ8 P2, respectively (Fig. 1). Although no solution was found with PHASER when using φ6 P2 as a search model, a possible solution could be obtained with φ12 P2 (TFZ: 9, eLLG:44) which is in line with the higher sequence identity between the φ8 and φ12 proteins. However, the AF2 model produced a definitive solution with TFZ = 20.3 and eLLG = 226 which indicated a more complete model.

**Fig. 2:**
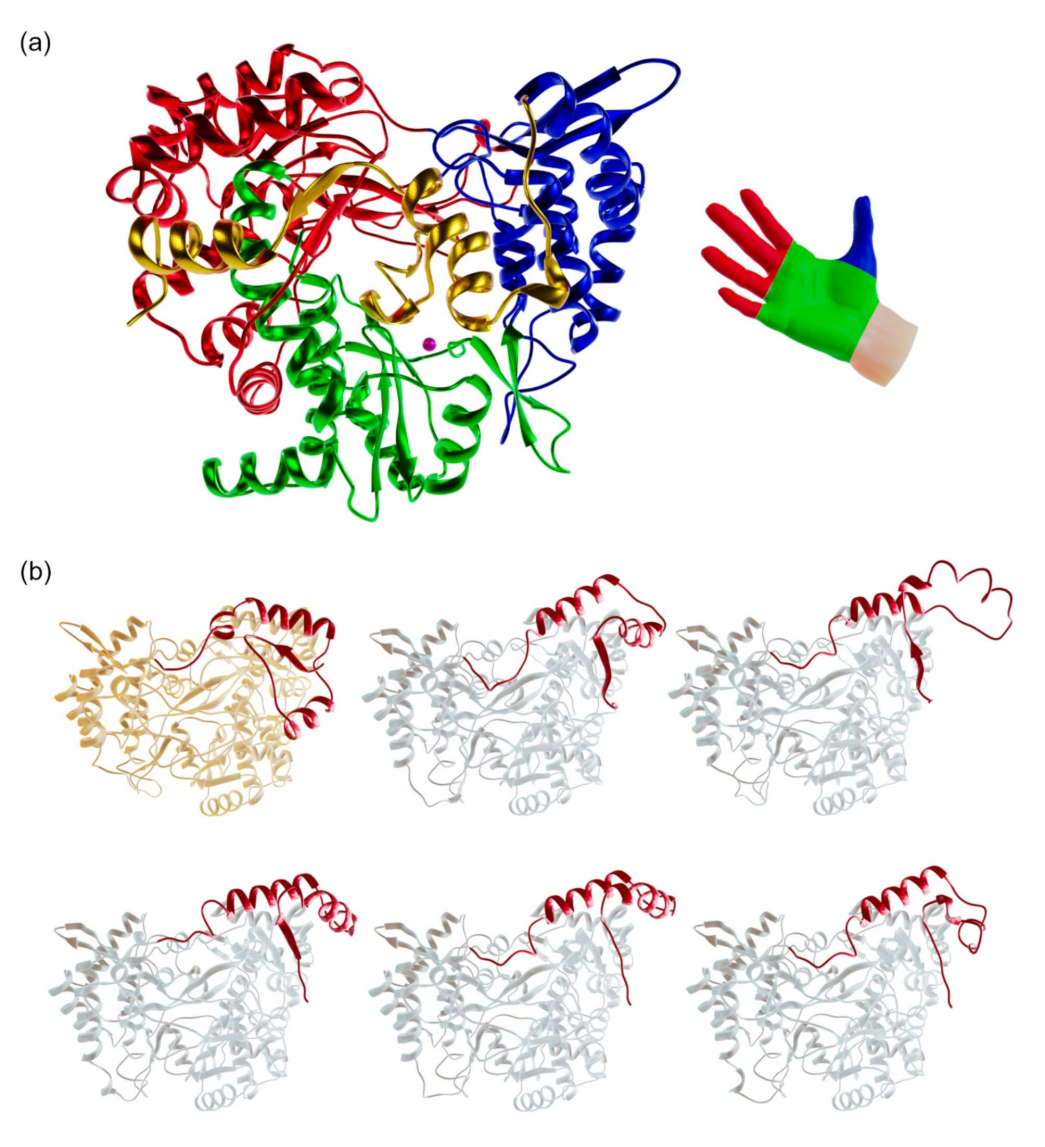
Structure of φ8 P2 and AF2 predictions. (**a**) Cartoon representation of the crystal structure of φ8 P2 showing the fingers, palm, thumb and C-terminal subdomains coloured in red, green, blue and yellow respectively. The bound Ca^2+^ ion is shown as a magenta sphere. (**b**) Comparison of the refined φ8 P2 model coloured in yellow and the five AF2 predicted models coloured in grey. The first 50 residues of N-terminal subdomains of each model are coloured in red to highlight the structural differences.

The overall structure of φ8 P2 is roughly globular, consisting of 24 α-helices and 19 β-strands (Figs. 1 and 2a). It has a typical RNA polymerase fold and resembles a cupped right hand with finger, thumb and palm subdomains, which together form the active site of the enzyme. The palm subdomain exhibits a structural arrangement consisting of six α-helices and seven β-strands. It stands out as the most conserved subdomain when comparing various viral polymerases, perhaps unsurprisingly since it houses the catalytic active site^28^. In contrast, the thumb subdomain, composed of five α-helices and three β-strands, and the finger subdomain composed of ten α-helices and seven β-strands are more variable.

Alterations in the relative orientations of the finger and thumb subdomains are closely linked to conformational shifts of the polymerase throughout various replication stages^28^. Like in P2 polymerases from φ6^7^ and φ12^18^, φ8 P2 thumb and finger subdomains establish a connection through the fingertips. This arrangement gives rise to a narrow channel that effectively restricts the passage of dsRNA, while allowing the ssRNA to traverse through^1^. It must be noted that the first 50 residues of the finger subdomain were not precisely predicted by any of the five AF2 models (Fig. 2b). This is possibly due to the lack of similar protein sequences to create a robust multiple sequence alignment. AF2 provided an improved molecular replacement search model, but as described elsewhere, it is a valuable hypothesis that does not replace experimental models^29^. These N-terminal residues complement the finger subdomain and fold as a β-strand (forming a β-sheet in conjunction with β12 and β13), followed by three α-helices.

Comprising three α-helices and one β-strand, the φ8 P2 C-terminal subdomain covers the catalytic cleft formed at the interface of the fingers and the thumb subdomains (coloured in yellow in Fig. 2a) and obstructs the pathway through which the nascent dsRNA would emerge, implying the need for a conformational modification to facilitate the release of the elongating chain as suggested for φ6 P2^7^. In other RdRps, the C-terminal size can vary significantly. For example, the C-terminal domain of the rotavirus RdRp VP1 consists of about 310 residues and is still anticipated to perform a similar function^30^. In cystoviruses, the C-terminal subdomain assumes an additional role as a priming platform. This enables the de novo synthesis of RNA on a ssRNA template, eliminating the requirement for a primer^7^. It is worth noting that this domain is absent in RdRps that require a primer, such as those found in picornaviruses, which feature larger central cavities^31^.

### Similarities and differences with φ6 and φ12 P2 RdRps

**Fig. 3:**
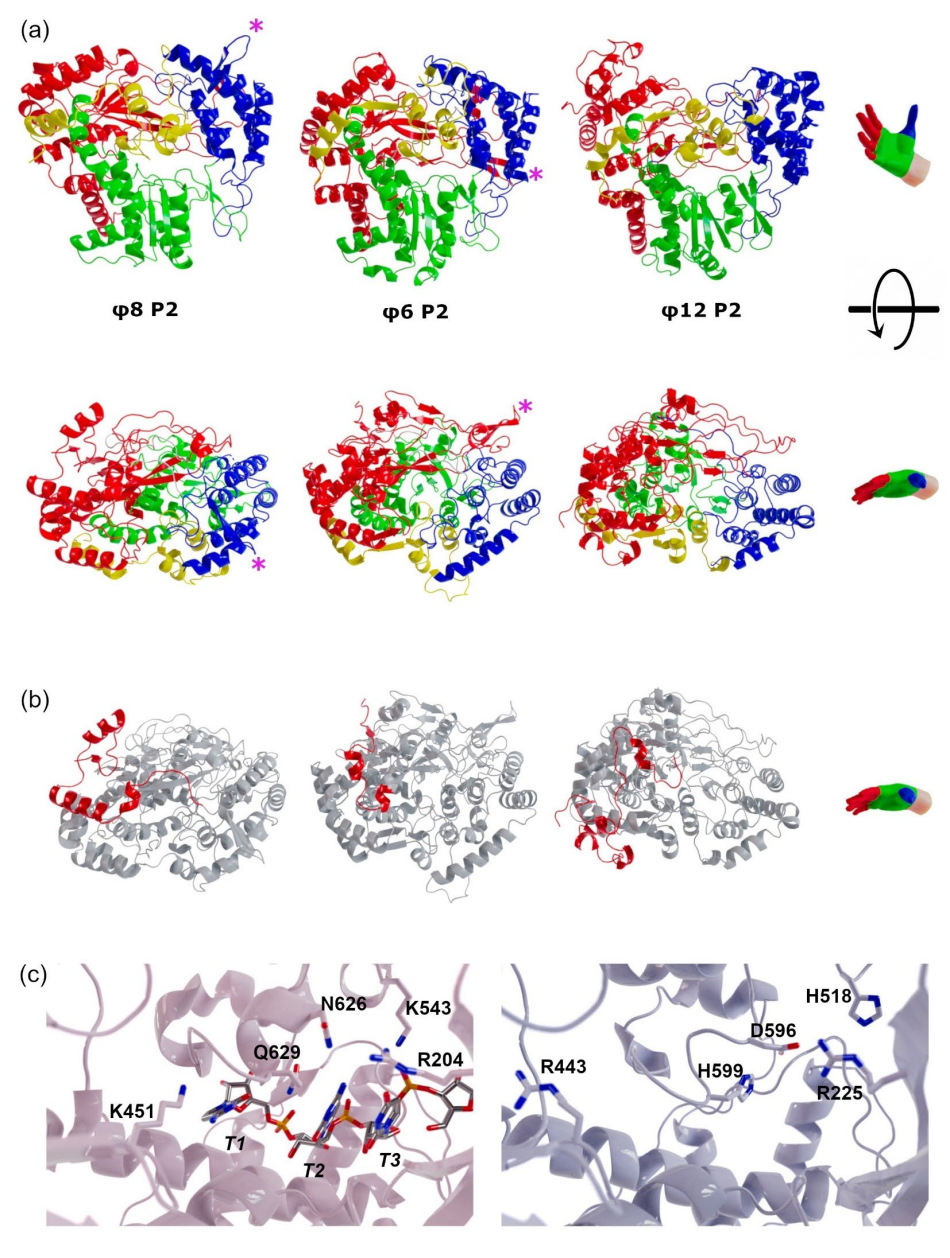
X-ray structures of cystoviral RdRps. **(a)** The crystal structures of φ8 P2, φ6 P2 (PDB: 1HHS) and φ12 P2 (PDB: 4GZK) proteins are shown in cartoon representation with the fingers, palm, thumb and C-terminal subdomains coloured in red, green, blue and yellow respectively. The pink asterisks indicate the location of the β-hairpin found in φ8 P2 and φ6 P2. **(b)** Comparison of the N-terminal of φ8 P2, φ6 P2 and φ12 P2. The first 50 residues of φ8 P2 and equivalent residues in φ6 P2 and φ12 P2 are coloured in red while the rest of the proteins are coloured in grey. **(c)** ssRNA binding site of φ6 P2 on the left (PDB: 1UVJ) and φ8 P2 on the right. Proteins are shown in cartoon representation and side chains and RNA in sticks. Oxygen, nitrogen and phosphorous atoms are coloured in red, blue and orange respectively. Carbon atoms from φ6 P2, RNA and φ8 P2 are coloured in pink, silver and grey respectively.

Despite the low sequence identity, the overall fold of φ8 P2 is similar to that of φ6 P2 (Root mean square deviation (RMSD) of the Cα atoms over 540 residues: 2.1Å) and φ12 P2 (RMSD of the Cα atoms over 534 residues: 2.1Å) and displays all the conserved structural features of RdRps. (Figures 1 and 3). The main difference between φ8 P2 and the other two P2 structures appears to be in the first 50 residues at the N-terminus (Fig. 3b). Significant variation in sequence (Fig. 1) and structure (Fig. 3) can be observed in the N-terminus among the three polymerases, likely to suit the unique requirements and characteristics of each viral entity. In the cryo-electron microscopy (cryoEM) localised reconstruction of φ6 P2^3^, the N-terminus is exposed to the inside of the capsid, suggesting an interaction with the packaged single-stranded viral genome.

Within the thumb subdomain, a notable variation is observed where a compact loop consisting of four residues in φ6 and φ12 P2, is substituted with an elongated β-hairpin structure spanning eleven residues (β2-β3) in φ8 P2. Intriguingly, a similar-length β-hairpin is present in φ6 P2 polymerase (residues 211–222), albeit situated in the finger subdomain. Both β-hairpins are exposed to the surrounding solvent or nucleic acid in the context of the capsid (Fig. 3), underscoring their potential functional significance within their respective subdomains, although it should be noted that φ12 P2 does not harbour such β-hairpin structure.

## Template specificity

During initiation of polymerization, φ6 P2 can bind diverse templates but shows a preference for ssRNA templates with terminal cytosines, such as CC-3’ and CCC-3’. In contrast, φ8 P2 is not only stimulated by terminal cytosines but also initiates more efficiently with 3’-proximal uridines, such as GAAAAUUUC-3’, GUUUUUUCC-3’, UC-3’, and U-3’^19^.

φ6 P2 has been crystallised with ssDNA and ssRNA templates^7,8,10^, ssDNA showed a different mode of binding compared to the physiological substrate ssRNA, due to the ribose hydroxyl group interactions with the protein^9^. The 3’ cytosine, named T1, is buried in the S site, a pocket past the catalytic site that is too narrow to accept a purine base^7^. T1 hydrogen bonds with the main chain carbonyl group of Q629, the side chain of K451. The T2 cytosine establishes hydrophobic interactions with residues R291 and A272 and base stacks with the uracil in position T3, which forms hydrogen bonds with G275, M273, R204, and K543^9^.

In φ8 P2, the only residue that could discriminate pyrimidine bases in the T1 position is R443 (K451 in φ6 P2), but arginine and lysine residues can both contact the N4 nitrogen of cytidine or the O4 oxygen of uracil bases, so the distinction between pyrimidines is not clear from the crystal structures of φ6 and φ8 P2. The same could be said for the T2 and T3 positions, where the only residues interacting with the T2 and T3 bases are R204 (R225 in φ8 P2), N626 (D596 in φ8 P2), S393 (S388 in φ8 P2). Based on the available structural data it is not evident how these interactions would distinguish between uridine and cytidine. An important feature is the Q629 residue located between the T1 and T2 bases and hydrogen bonding T1 cytosine base which is replaced in φ8 P2 by H599. The histidine side chain would allow π-stacking interaction with T2 and a better stabilisation of T2 and T3 bases. Similarly, in φ12 P2, a residue with an aromatic side chain from tyrosine is also found at this position. The available crystal structures of φ6 and φ8 P2 do not give insights into the subtle selection between pyrimidine bases, so structures of the polymerases with different ssRNA templates would help to further understand the specificity.

## Metal ion binding site-

The φ6 P2 polymerase possesses two distinct metal ion binding sites: a non-catalytic site that can be occupied by either a Mn^2+^ or Mg^2+^ ion, and a catalytic site consisting of two Mg^2+^ ions^7^. It is noteworthy that the residues involved in coordinating the catalytic ions demonstrate significant conservation, as observed in cystoviral polymerases (Fig. 1), and even among unrelated viruses like hepatitis C virus (HCV)^7,32^. Mn^2+^increases the specific activity of φ6 P2 by more than an order of magnitude^33^, and similar stimulatory effects have been described for a number of viral RdRps, including those from HCV, poliovirus, reovirus^34–36^. The mechanism of this stimulation is still poorly understood.

The non-catalytic ion site also named metal site B’, is involved in regulating the activity of the polymerase and is mostly found in viral polymerases structures^37^. It is different from the third metal C initially characterised in the human DNA polymerase η^38^. Binding specific metal ions at the metal site B’ site can modulate the function of the enzyme by influencing its stability^10^.

Because of the high concentration of CaCl_2_ in the crystallisation condition, a Ca^2+^ ion was modelled at the non-catalytic ion site of the φ8 P2 structure. Ca^2+^ions have been previously detected in the non-catalytic ion binding sites of other RdRps^32^. To rule out the presence of other metal ions that could have been picked up during protein expression or purification, the identification of the Ca^2+^ion has been confirmed using f’’ refinement. A common use of anomalous scattering is for ion identification, while this typically involves calculating Fourier anomalous difference maps, a possible alternative is through f’’ refinement^39^. This utilises the principle that f’’ varies with wavelength for a single element. For data taken at a given wavelength, f’’ can be refined against the X-ray data and compare this value to measured or tabulated theoretical values. A result close to the theoretical f’’ value of calcium at λ = 0.93Å (0.5 e^−^) confirms the Ca^2+^ ion identity.

**Fig. 4:**
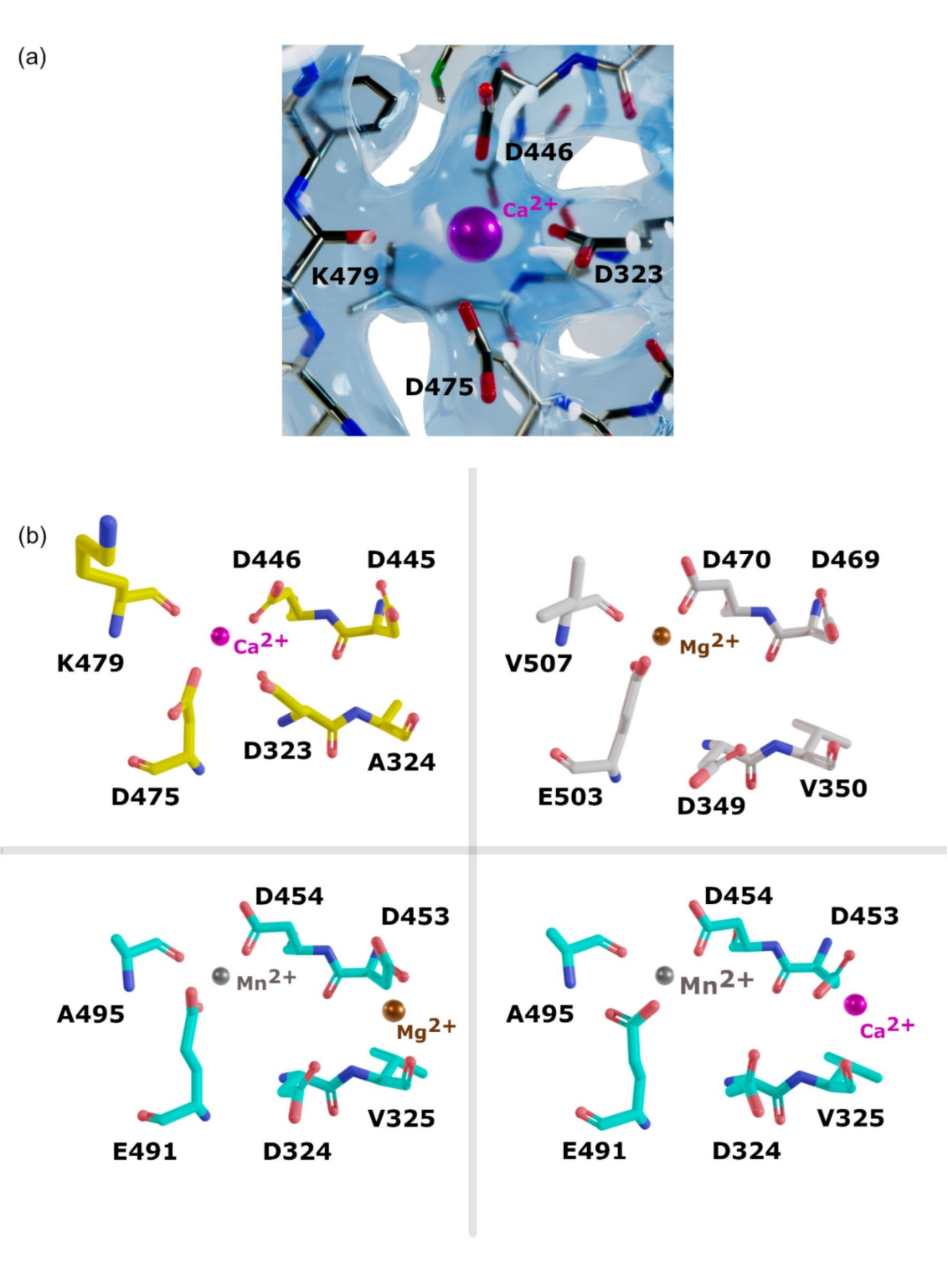
Metal ion binding sites in cystoviral RdRps. **(a)** Non-catalytic ion binding site of φ8 P2. Ca^2+^ is shown as a magenta sphere and protein residues as sticks. 2fo-fc electron density is contoured at 1 σ and shown as transparent surface. **(b)** Non-catalytic and catalytic metal ion binding sites of φ8 P2 (carbon coloured in yellow, top left), φ12 P2 (carbon coloured in grey, top right, PDB: 4IEG), φ6 P2 (carbon coloured in cyan, bottom left, PDB code: 1HI0; bottom right, PDB code: 1UVN)). Calcium, manganese and magnesium ions are shown as spheres and coloured in magenta, grey and brown respectively.

The Ca^2+^ ion is coordinated by the sidechains of three aspartate residues (D323, D446, D475) as well as the main-chain carbonyl of lysine K479 (Fig. 4a). Interestingly, D323 is found in a conformation pointing away from the catalytic site. The Ca^2+^ ion is situated in a comparable location to the Mn^2+^ found in the non-catalytic site of φ6 P2 (PDB code: 1HHS), however, in φ6 P2, the Mn^2+^ ion is coordinated only by three residues: D454, E491, and the main-chain carbonyl of A495 (equivalent to φ8 P2 residues D446, D475 and K479 respectively) (Fig. 4b). In φ6 P2, the aspartic acid residue D324, equivalent to φ8 P2 D323, is found in a conformation with its side-chain pointing toward the active site and, along with V325 and D453, participates in the formation of the adjacent catalytic ion site^10^ (Fig. 4b). Both aspartate residue conformations are seen in other RdRp structures such as in the poliovirus structures (PDB open 3OL6, close, 3OL7) and in hepatitis C virus RdRp (PDB open 2XI2, close, 4WTA), and are termed open and closed conformations^40,41^ where the motif A aspartate reorients from the metal site B’ to the metal site B during active site closure. It was suggested that this reorientation facilitates the movement of the bound ion toward the catalytic site^37^. This implies that φ8 P2 D323 contributes to the coordination of both the non-catalytic and catalytic ion sites and that the structure reported here corresponds to an open conformation. In the poliovirus RdRp, open and close structures, hydrogen bonds are formed between anti-parallel β-strands between motifs A and C, but in the open state the β-sheet is frayed near the active site^37^. In φ8 P2, although D323 is in an open conformation, the motifs A and C β-sheet is not frayed near the catalytic site.

The metal site B’ is expected to be occupied by a metal ion, such as Mg^2+^ or Mn^2+^, that can activate the polymerase. In φ8 P2, the presence of Ca^2+^ ions could lock D323 into an inactive conformation, coordinating the non-catalytic site instead of the catalytic site, that hinders its ability to catalyse the reaction.

Previous studies have demonstrated that Ca^2+^ ions inhibit the φ6 replication and transcription by disrupting the initiation complex and replacing the catalytic ions (Fig. 4b), thus stalling the reaction^9,42,43^. The structure presented in this study also reveals an inactive conformation in the presence of Ca^2+^ ions. However, it remains unclear whether this conformation is an artifact of crystallization or a mechanism of inactivation under high concentrations of CaCl_2_.

### φ8 P2 polymerase and φ8 P1 procapsid proteins interactions

**Fig. 5:**
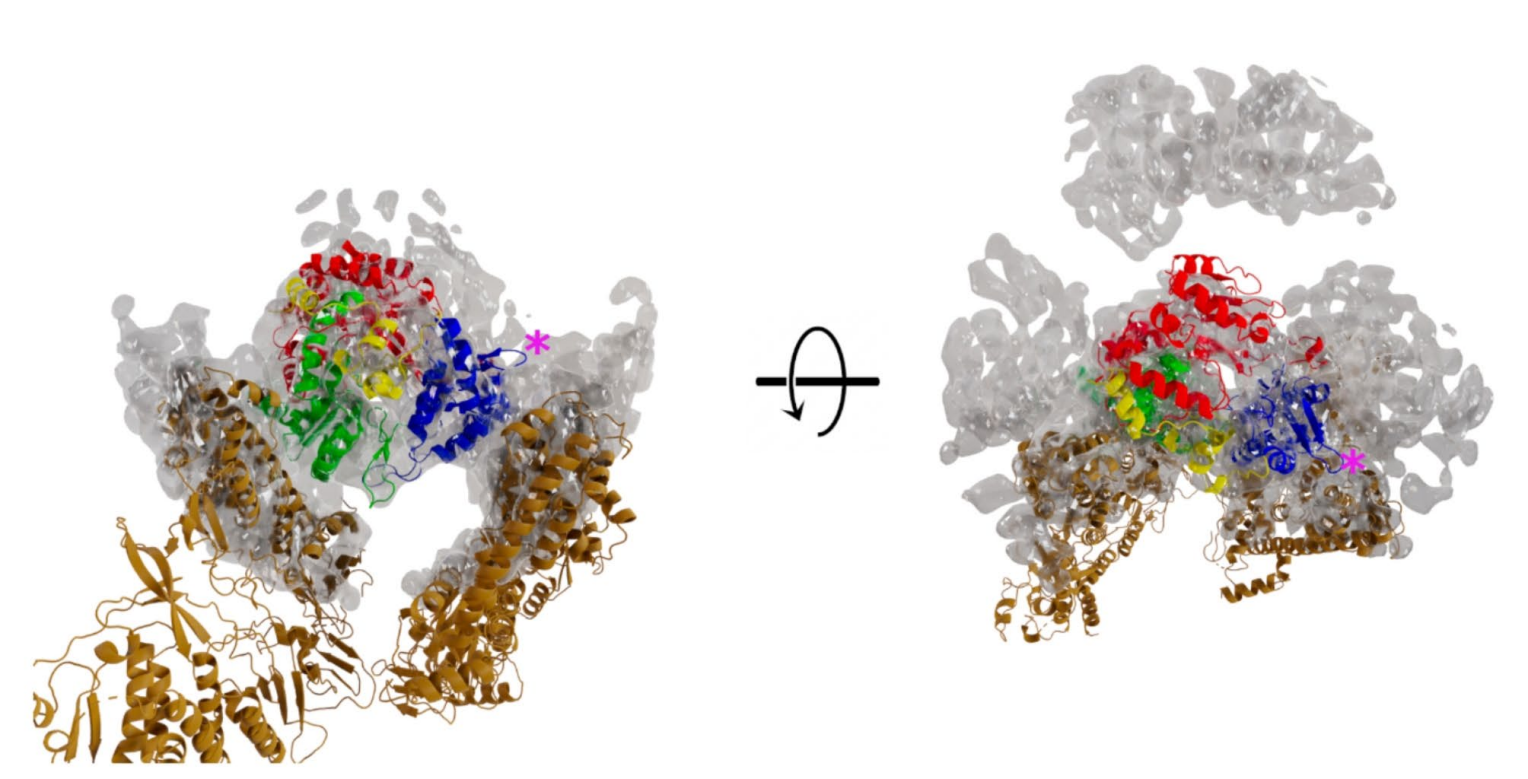
Fitting of φ8 P2 in the φ6 procapsid cryo-EM reconstruction. Localised reconstruction of φ6 P2 in the procapsid at 7.9Å resolution (EMD-3185) fitted with the X-ray crystal structures of φ8 P2 coloured as in Fig. 2a, and φ8 P1 (PDB code: 4BX4) coloured in dark orange. The pink asterisks indicate the location of the β-hairpin.

By employing a 7.9Å resolution cryoEM localised reconstruction of the φ6 P2 density from the in vitro self-assembly system of φ6 polymerase complexes it was possible to accurately position the φ6 P2 RdRp at the three-fold axis within the φ6 procapsid^3^. Given the remarkable similarity between the structures of bacteriophages φ6 and φ8^6,44,45^, φ8 P1 and P2 proteins were superimposed onto their φ6 counterparts and fitted into the localized reconstruction of the φ6 procapsid (Fig. 5). Similar to the φ6 P1-P2 interaction, two contact sites can be identified, involving the palm subdomain and thumb subdomains, respectively. Due to the limited resolution of the reconstruction and the local structural differences between φ6 and φ8 P1 and P2 proteins, it is not possible to determine the exact residues involved in the interaction. Nevertheless, a number of polar and charged residues are found at both interfaces. The β-hairpins found in φ6 and φ8 P2 (Fig. 3) are not interacting with P1 proteins, but instead exposed on the surface to interact with the RNA genome or the P7 protein (Fig. 5). The loops interacting with the capsid protein P1 or the genomic RNA represent unique structural adaptations of cystoviral polymerases, enabling them to function efficiently within the crowded viral capsid environment.

In conclusion, this study presents the X-ray structure of φ8 P2 at 3Å resolution. While sharing similarities with φ6 and φ12 RdRp structures, this study reports a distinct N-terminal subdomain that could not be predicted by AF2. Moreover, a Ca^2+^ ion is present in the non-catalytic site instead of Mn^2+^ or Mg^2+^ ions as observed in φ6 and φ12 polymerases structures. When aspartate D323 coordinates with the Ca^2+^ ion, it can disrupt or prevent coordination with the catalytic Mg^2+^ ion. This alteration in conformation may indeed contribute to the inhibitory effect observed for φ6 P2.

## Data Availability

The structure factors and coordinates for φ8 P2 have been deposited in the Protein Data Bank (https://www.rcsb.org/) under the accession code 9FEJ.
